# Effect of changes in treatment practice on survival for cervical cancer: results from a population-based study in Manitoba, Canada

**DOI:** 10.1186/s12885-015-1624-z

**Published:** 2015-09-22

**Authors:** Yoon-Jung Kang, Dianne L. O’Connell, Robert Lotocki, Erich V. Kliewer, David E. Goldsbury, Alain A. Demers, Karen Canfell

**Affiliations:** 1 Prince of Wales Clinical School, the University of New South Wales, Sydney, NSW Australia; 2 Cancer Research Division, Cancer Council NSW, 153 Dowling Street, Woolloomooloo, NSW Australia; 3 Division of Gynecologic Oncology, CancerCare Manitoba, Winnipeg, MB Canada; 4 Epidemiology and Cancer Registry, CancerCare Manitoba, Winnipeg, MB Canada; 5 Community Health Sciences, University of Manitoba, Winnipeg, MB Canada

## Abstract

**Background:**

Results from clinical trials in the 1990s led to changes in the recommended treatment for the standard therapy for stage IIB-IVA cervical cancer from radiotherapy alone to chemo-radiotherapy. We conducted the first population-based study in Canada to investigate temporal treatment patterns for cervical cancer and long-term survival in relation to these changes in the treatment guidelines.

**Methods:**

Detailed information on stage and treatment for 1085 patients diagnosed with cervical cancer in 1984–2008 and identified from the population-based Manitoba Cancer Registry (MCR) in Canada was obtained from clinical chart review and the MCR. Factors associated with receiving guideline treatment were identified using logistic regression. All cause and cervical cancer specific survival were compared in patients who were and were not treated as recommended in the guidelines, using Cox proportional hazards models.

**Results:**

The median follow-up time was 6.4 years (range: 0.05–26.5 years). The proportion of women who received guideline treatment was 79 % (95 % confidence interval [CI]: 76–81 %). However, the likelihood of being treated according to the guidelines over time was modified by age (*p* < 0.0001) and tumour stage at diagnosis (*p* = 0.002). Women who were treated according to the guidelines after the change in recommended clinical practice (1999–2008) had a significantly lower risk of death from all causes and from cervical cancer. This was driven by lower mortality rates in cases with stage IIB-IVA tumours (all causes of death: hazard ratio [HR] = 0.60, 95 % CI: 0.43–0.82, *p* = 0.002; cervical cancer related death: HR = 0.64, 95 % CI: 0.44–0.93, *p* = 0.02).

**Conclusions:**

The management of cervical cancer patients in Manitoba, Canada was in good agreement with treatment guidelines although reasons for departure from the guideline recommendations could not be examined further due to lack of data. Treatment of stage IIB-IVA cervical cancers with recommended concurrent chemo-radiotherapy, which is now standard practice, was associated with substantially increased survival, although the effect of changes in clinical practice including maintenance of haemoglobin levels on improved survival cannot be ruled out as a contributing factor.

## Background

Until the 1990s the standard therapy for *International Federation of Gynecology and Obstetrics* (FIGO) stage IIB-IVA cervical cancer, or earlier stage disease with adverse pathological features, involved radiation alone. However, a rapid increase in concurrent use of chemo-radiotherapy has occurred since the mid-1990s, after multi-centre randomised controlled trials (RCTs) [1–3] found cisplatinum-based concurrent chemo-radiotherapy prolonged survival in patients with advanced cervical cancer compared to radiotherapy alone. Subsequently, treatment guidelines in many jurisdictions [4–7] incorporated this new evidence. By contrast, the recommended treatments for early stage disease (FIGO Stage I-IIA), consisting of surgery with or without adjuvant radiotherapy, have not changed substantially over the last few decades. In Canada, guidelines for cervical cancer management have not been formulated at a national level, but the available provincial guidelines in Ontario [8, 9] and British Columbia [10] do not substantially differ from the guidelines developed by the FIGO [11, 12] or available guidelines in other countries [4, 5, 7]. Therefore, “synthesised” guidelines, derived from available Canadian provincial and international guidelines, reflecting the available evidence can be readily formulated for Manitoba.

Studies from two Canadian centres in Ontario have investigated trends in the use of concurrent chemo-radiotherapy and resulting improved survival outcomes in cervical cancer patients, without adjusting for tumour stage [13, 14]. However, a population-based study investigating survival outcomes with long term follow up in women who were and were not managed in concordance with treatment guidelines has not previously been performed in the Canadian setting. Therefore, the aims of this study were to describe: 1) trends in treatment patterns in relation to changes in guideline recommendations; 2) the proportion of cervical cancer patients receiving treatment as recommended in the guidelines; 3) factors related to receiving treatment according to the guidelines; and 4) the impact of adhering to guidelines on the risk of death from all causes (i.e., any death) and from cervical cancer in the Canadian province of Manitoba.

## Methods

### Study sample and data sources

The population-based Manitoba Cancer Registry (MCR) was used to identify all incident cervical cancer cases diagnosed over the period 1984 to 2008 [15]. More detailed information on treatment was obtained by combining the MCR and a database derived from chart reviews (available only for the years 1984–1999); the registry and the charts are both maintained by CancerCare Manitoba.

Treatment procedures were coded using ICD-9-CM Volume 3 from 1984 to 2004 and the Canadian Classification of Health Interventions from 2005 to 2008: these two classification systems are comparable [16]. Morphologic data were coded using ICD-O-2 (1984–2000) and ICD-O-3 (2001–2008) that were comparable to each other. Cause of death was coded using ICD-9 until 1999 and ICD-10 thereafter. Although comparability between ICD-9 and ICD-10 on cause of death could not be examined for the current dataset, it was reported that there was a 2 % increase in cervical cancer death when using ICD-10 compared to using ICD-9 [17].

Information on patients’ performance status, comorbidities and recurrence were not recorded on either clinical chart or the MCR. Disease stage was defined according to the FIGO staging system (1984–1999) and equivalent American Joint Committee on Cancer (AJCC) staging system (2004–2008). For those who were diagnosed in 2000 to 2003, a stage based on the agreement between FIGO stage and clinical TNM category was used. This was based on the fact that the agreement between FIGO stage and clinical TNM staging, using the clinical chart review dataset that contains both staging information, was substantial (kappa = 0.74, weighted kappa = 0.83) [18]. The agreement between the two staging systems was relatively lower for patients with stage IB2-IIA disease (68 %), but the proportions under-staged or over-staged were similar (15 % vs 18 %, respectively).

During 1984 to 2008, a total of 1413 incident cases of cervical cancer were identified from the MCR. For the overlapping period 1984 to 1999, the reliability of the two data sets was examined by comparing seven indicators including the number of cases diagnosed in each year, date of diagnosis, age at diagnosis, treatment procedures and related dates, histology and cause of death. During the period, the total number of patients identified from any of the two data sets was 1043. Of these, 845 (81 %) were found in both data sets, and the remaining number of patients included 87 non-residents in the clinical chart review and 111 residents in the clinical cancer registry. For the 845 patients identified in both data sets, there was full agreement for six out of the seven indicators. The only exception was cause of death. For the time period 1984 to 1999, the MCR was used to determine the vital status if the information in the MCR and the chart review was inconsistent (12 out of 845 patients). For 328 patients there was either no tumour stage information and/or they received no treatment: 264 patients had no tumour stage information; 53 patients had no treatment records and it was not possible to identify whether they did or did not receive any treatment; for 11 patients neither FIGO stage nor treatment records were available. The final study sample consisted of 1085 (77 %) cervical cancer cases. There were no differences in demographic and clinical characteristics for those included and not included in the analysis (results not shown).

### Treatment recommendations in the guidelines

As there are no published national guidelines for cervical cancer treatment in Canada, synthesised guidelines were derived from available provincial (published 2002 onwards) and international (FIGO) consensus and evidence-based treatment guidelines. Although changes occurred over time, there were no substantial differences identified between the provincial and the FIGO guidelines. For the purpose of the analysis, the FIGO guidelines were used as reference to evaluate the clinical practice in Manitoba for the years 1984 to 1998 [12, 19]. For 1999 to 2008, the synthesised evidence-based guidelines were used (Table 1) [4–7, 11]. It was not possible to determine if the course of the treatment was completed or if the treatment schedule/doses were modified due to intolerance or choices by physicians and/or patients, due to data availability. Similarly, the exact timing and mechanisms for the guideline implementation in the local setting were not available from the administrative data.

**Table 1:** Synthesised guidelines for treatment of cervical cancer cases

	Recommended treatment	
FIGO stage	Consensus guidelines^a^ [12, 19] (applicable to 1998)	Synthesised evidence-based guidelines [4–7, 11] (applicable from 1999 onwards)
IA1	Total hysterectomy, conisation, radical hysterectomy^b^, radiotherapy^c^	Total hysterectomy, conisation, radiotherapy
IA2	Radical hysterectomy, total hysterectomy, radiotherapy^c^	Radical hysterectomy, total hysterectomy^d^, trachelectomy, radiotherapy^c^
IB-IIA		
*≤4 cm*	Radical hysterectomy, radiotherapy	Radical hysterectomy, radiotherapy,
		Radical hysterectomy + adjuvant radiotherapy (chemo-radiotherapy)
*>4 cm*	Radical hysterectomy, radical hysterectomy + adjuvant radiotherapy	Radical hysterectomy, chemo-radiotherapy,
		Radical hysterectomy + adjuvant radiotherapy(chemo-radiotherapy)
IIB-IVA	Chemo-radiotherapy, radiotherapy	Chemo-radiotherapy
IVB	Radiotherapy (curative/palliative), chemotherapy	Radiotherapy (curative/palliative), chemotherapy, chemo-radiotherapy

^a^Development of consensus guidelines is a long process and we assumed that the evidence supporting the decision was available before the guidelines were published. Therefore, we measured concordance up to 1998 based on consensus guidelines published up to 2000

^b^Radical hysterectomy was used if there was lymph-vascular permeation on the cone biopsy

^c^Radiotherapy was used if medically inoperable

^d^Total hysterectomy was used if there was no lympho-vascular permeation on the cone biopsy

### Statistical analysis

**Fig. 1:**
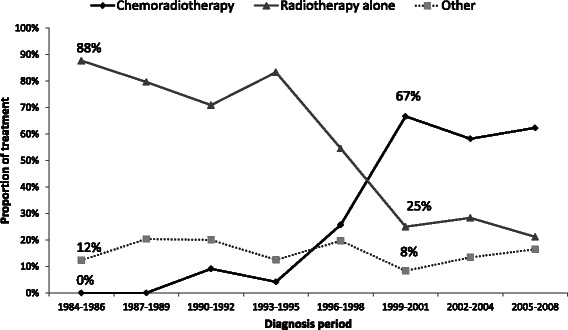
Trends in the initial treatment for cervical cancer patients diagnosed with stage IB2-IVA tumours (n = 513). Other treatment includes surgery alone, chemotherapy alone, pre-operative radiation followed by surgery, surgery with adjuvant chemotherapy, surgery with adjuvant radiotherapy, palliative radiation and no treatment

The overall trend in the initial treatment during 1984 and 2008 was described using the 3-year average (Fig. 1). Treatment patterns for cervical cancer patients by diagnosis period (1984–1998, 1999–2008) stratified by tumour stage (IA, IB-IIA, IIB-IVA and IVB) were cross-tabulated. Bivariable analyses were conducted to examine differences in demographic and tumour characteristics of women who did and did not receive treatment recommended in the guidelines.

A binomial logistic regression model was fitted to identify the factors associated with receiving guideline treatment (i.e., treatment according to the guidelines). Factors examined included tumour stage; age at diagnosis (0-45 years, 46–65 years, >65 years); diagnosis period (1984–1998, 1999–2008); histology (squamous cell carcinoma or adenosquamous carcinoma, adenocarcinoma or other histology); and area of residence (urban [Winnipeg and Brandon], rural).

A Cox proportional hazards regression model was used to examine the association between receiving the treatments as recommended and the risk of death from all causes and from cervical cancer. Time to death was calculated from the date of diagnosis to the date of death or censored at 30 June 2010. Potential confounders included diagnosis period, age, histology, area of residence and tumour stage. Stratified analysis by tumour stage was also performed. Data were analysed using SAS 9.2 (SAS Institute Inc., Cary, NC, USA).

### Ethics approval

The study obtained human research ethics approval from the University of Manitoba Health Research Ethics Boards, the University of Sydney Human Research Ethics Committee and Cancer Council NSW Human Research Ethics Committee. As this study used de-identified data, all Human Research Ethics Committees waived the need for consent to participate in this study.

## Results

### Baseline characteristics

The median age at diagnosis of invasive cervical cancer was 50 years (range: 16–89). The majority (74 %) of patients were diagnosed with either stage IB to IIA or IIB to IVA disease. The proportion of women over 65 years of age was greater in those diagnosed with stage IIB-IVB disease than those with stage IA-IIA disease (32 % and 14 %, respectively). The majority of patients lived in urban areas at the time of diagnosis (65 %). Squamous cell carcinoma (including adenosquamous carcinoma) was the most common histology type (80 %) (Table 2). Surgery alone was the most frequently used treatment for patients with IA and IB-IIA stage disease (93 % and 44 %, respectively), whereas radiotherapy alone was the most frequently used therapy for patients with IIB-IVA and IVB stage disease (60 % and 73 %, respectively) (Table 3).

**Table 2:** Characteristics of cervical cancer cases diagnosed in 1984–2008 by adherence to treatment guidelines (*n* = 1085^a^)

	Treated according to treatment guidelines			
	Total	Yes (*n* = 852)	No (*n* = 233)	
Characteristics	No. (Column %)	No. (Row %)	No. (Row %)	*p*-value*
Tumour stage				0.005
IA	230 (21)	175 (76)	55 (24)	
IB-IIA	400 (37)	301 (75)	99 (25)	
IIB-IVA	435 (37)	326 (75)	109 (25)	
IVB	52 (5)	50 (96)	2 (4)	
Age at diagnosis				0.22
0–45	518 (48)	398 (77)	120 (23)	
46–65	333 (31)	261 (78)	72 (22)	
>65	234 (22)	193 (82)	41 (18)	
Histology				0.04
SCC	866 (80)	691 (80)	175 (20)	
Others	219 (20)	161 (74)	58 (26)	
Area of residence				0.90
Urban	707 (65)	554 (78)	153 (22)	
Rural	378 (35)	298 (79)	80 (21)	
Diagnosis period				<.0001
1984–1998	718 (66)	594 (83)	124 (17)	
1999–2008	367 (34)	258 (70)	109 (30)	

*SCC* squamous cell carcinoma/adenosquamous carcinoma

*For chi-square test of association

^a^After excluding cases with either missing stage data (14 %, mostly diagnosed in 2000–2003), cause of death (4 %) or treatment records (5 %)

**Table 3:** Treatment administered to cervical cancer cases by tumour stage and time period (1984–1998 and 1999–2008) (*n* = 1085)

	Women treated for cervical cancer according to the guidelines by tumour stage and time period															
	Stage IA^a^ (*n* = 229)				Stage IB-IIA^b^ (*n* = 398)				Stage IIB-IVA^c^ (*n* = 406)				Stage IVB (*n* = 52)			
	1984–1998		1999–2008		1984–1998		1999–2008		1984–1998		1999–2008		1984–1998		1999–2008	
Treatment	No./Total	%	No./Total	%	No./Total	%	No./Total	%	No./Total	%	No./Total	%	No./Total	%	No./Total	%
Surgery alone	121/152	80	46/62	74	95/138	69	32/36	89	0/3	0	0/2	0	0/0	-	0/0	-
Surgery + adjuvant radiotherapy	0/7	0	0/0	-	32/44	73	8/14	57	0/2	0	0/9	0	0/0	-	0/1	0
Preoperative radiotherapy + surgery	0/0	-	0/0	-	5/7	71	0/2	0	0/14	0	0/0	-	0/0	-	0/0	-
Surgery + adjuvant chemotherapy	0/0	-	0/0	-	0/2	0	0/0	-	0/0	-	0/0	-	0/0	-	0/1	0
Radiotherapy alone	7/7	100	1/1	100	99/99	100	8/9	89	196/196	100	0/49	0	17/17	100	21/21	100
Chemo-radiotherapy	0/0	-	0/0	-	0/6	0	22/38	58	22/22	100	108/108	100	0/0	-	10/10	100
Chemotherapy alone	0/0	-	0/0	-	0/2	0	0/1	0	0/0	-	0/1	0	0/0	-	2/2	100
Total	128/166	77	47/63	75	231/298	78	70/100	70	218/237	92	108/169	64	17/17	100	33/35	94
% (95 % CI) of women treated according to the guidelines																
By tumour stage and time period	77 % (70–83 %)		75 % (62–85 %)		78 % (72–82 %)		70 % (60–79 %)		92 % (88–95 %)		64 % (56–71 %)		100 % (80–100 %)		94 % (81–99 %)	
By tumour stage in 1984-2008	76 % (70–82 %) in 1984–2008				76 % (71–80 %) in 1984–2008				80 % (76–84 %) in 1984–2008				96 % (87–100 %) in 1984–2008			
By time period for all tumour stages	83 % (80–85 %) in 1984–1998, 70 % (65–75 %) in 1999–2008															
Overall	79 % (76–81 %) in 1984–2008															

*No.* number of women treated according to the guidelines

^a^Most patients who did not receive guideline treatment were treated with different surgery types (for example, LEEP with or without hysterectomy or total hysterectomy where radical hysterectomy was indicated or vice versa)

^b^Most patients who were not treated according to the guidelines were treated with total hysterectomy with or without adjuvant radiotherapy. Patients with bulky lesion and treated with chemo-radiotherapy were regarded as not receiving guideline treatment

^c^Patients diagnosed with advanced stage disease who received radiotherapy alone due to co-morbidities or poor performance status were regarded as not receiving guideline treatment

### Overall trends in the initial treatment

During the period 1984 to 2008, use of chemo-radiotherapy increased with a concomitant decrease in the use of radiotherapy alone, especially for patients with tumour staged IB2-IVA (Fig. 1). Until 1995, the predominant initial treatment (i.e., treatment given within the first year after diagnosis) for stage IB2-IVA tumours was radiotherapy alone. The use of combined chemo-radiotherapy started to increase steadily from 4 % in 1993–1995 and became the predominant treatment from 1999 onward (67 % of women).

### Guideline treatment

Most women with invasive cervical cancer in Manitoba received guideline treatment (79 %, 95 % CI: 76–81 %) over the study period. The overall proportion of women receiving guideline treatment was higher in 1984–1998 (83 %, 95 % CI: 80–85 %) than in 1999–2008 (70 %, 95 % CI: 65–75 %). The proportion of women receiving guideline treatment by tumour stage in the later period did not substantially differ from that in the earlier period, with the exception of stage IIB-IVA (92 % vs 64 %) where a substantial proportion of women received radiotherapy alone instead of chemo-radiotherapy in the later period (Table 3).

The bivariable analysis showed an association between receipt of treatment according to the guidelines and tumour stage (*p* = 0.005), tumour histology (*p* = 0.04) and time period (*p* < 0.0001) (Table 2). The effect of time period on the odds of receiving guideline treatment was modified by both stage (*p* = 0.002) and age at diagnosis (*p* < 0.0001) (Table 4). Compared with those who were diagnosed in 1984–1998, patients diagnosed with stage IA or IB-IIA tumours in 1999–2008 at over 65 years of age were significantly less likely to receive treatment according to the guidelines (OR = 0.11 in both stage groups). For patients diagnosed with stage IIB-IVA disease in the later period, women in all age groups were less likely to receive treatment according to the guidelines compared with those who were diagnosed in the earlier period.

**Table 4:** Factors associated with the probability of being treated according to treatment guidelines for cervical cancer cases diagnosed in 1984–2008 (*n* = 1033)

			Total no. cases	Adjusted OR (95 % CI)^a^	*p*-value
Histology					0.09
SCC			854	1.00	
Others			211	0.72 (0.50–1.05)	
Area of residence					0.77
Urban			691	1.00	
Rural			374	0.95 (0.69–1.32)	
Tumour stage^b^ by time period and age at diagnosis					
IA	0–45 years*	1984–1998**	119	1.00	
		1999–2008	50	0.99 (0.49–1.99)	
	46–65 years	1984–1998	35	1.00	
		1999–2008	11	0.97 (0.40–2.37)	
	>65 years	1984–1998	13	1.00	
		1999–2008	2	0.11 (0.04–0.33)	
IB–IIA	0–45 years	1984–1998	172	1.00	
		1999–2008	50	1.04 (0.57–1.90)	
	46–65 years	1984–1998	72	1.00	
		1999–2008	31	1.02 (0.50–2.08)	
	>65 years	1984–1998	56	1.00	
		1999–2008	19	0.11 (0.04–0.29)	
IIB–IVA	0–45 years	1984–1998	65	1.00	
		1999–2008	57	0.28 (0.13–0.57)	
	46–65 years	1984–1998	96	1.00	
		1999–2008	72	0.27 (0.14–0.54)	
	>65 years	1984–1998	104	1.00	
		1999–2008	41	0.03 (0.01–0.08)	

*OR* odds ratio, *SCC* squamous cell carcinoma/adenosquamous carcinoma

*Interaction between time period and age (*p* < 0.0001)

**Interaction between time period and tumour stage (*p* = 0.002)

^a^OR was adjusted for all variables shown in this table

^b^Patients with tumour stage IVB were not included in the analysis due to insufficient number

### Effect of guideline treatment on survival

The median follow-up time after diagnosis was 6.4 years (range: 0.05–26.50 years). The overall number of deaths due to cervical cancer and all causes was 312 and 473, respectively. Among patients diagnosed with stage IA disease and who were not treated according to the guidelines, there were no deaths from cervical cancer. Therefore, cervical cancer death probabilities were determined for those diagnosed with stage IB-IVB tumours only.

#### All-cause mortality

The risk of dying from any cause following a cervical cancer diagnosis increased with the stage of the disease and with increasing age at diagnosis (*p* < 0.0001) (Table 5). The effect of being treated according to the guidelines on all-cause mortality differed over the diagnosis period (*P*_*interaction*_ *= 0.0001*). Patients diagnosed in 1984–1998 and who were treated according to the guidelines had a similar risk of dying to those who did not (HR = 1.22, 95 % CI: 0.85–1.75). By contrast, women diagnosed 1999–2008 and who were treated according to the guidelines experienced a 56 % decreased risk of death from all causes (HR = 0.44, 95 % CI: 0.31–0.64). Histology (*p* = 0.47) and area of residence (*p* = 0.06) were not significantly associated with the risk of dying from all causes. The tumour stage stratified analysis (Table 6) showed that independent effects of being treated according to the guidelines as well as being diagnosed in the later period, were only observed for stage IIB-IVA disease, i.e., women who received concurrent chemo-radiotherapy had a significantly decreased risk of death from all causes (HR = 0.60, 95 % CI: 0.43–0.82, *p* = 0.002).

**Table 5:** Association between receipt of guideline treatment and the probability of dying for cervical cancer patients (all stage groups) who were diagnosed 1984–2008 (*n* = 1085)

		Any death			Death from cervical cancer		
		No. of death/Total	Adjusted HR (95 % CI)^a^	*p*-value	No. of death/Total	Adjusted HR (95 % CI)^a^	*p*-value
Tumour stage				<.0001			<.0001
IA^b^		23/229	1.00		1/229	-	
IB-IIA		139/398	3.47 (2.22–5.41)		80/398	1.00	
IIB-IVA		261/406	8.38 (5.39–13.03)		185/406	3.13 (2.36–4.15)	
IVB		50/52	68.09 (39.38–117.74)		46/52	25.60 (16.61–39.46)	
Age at diagnosis				<.0001			0.01
0–45		127/518	1.00		99/518	1.00	
46–65		165/333	1.69 (1.33–2.14)		106/333	1.12 (0.85–1.49)	
>65		181/234	2.64 (2.07–3.37)		107/234	1.52 (1.14–2.03)	
Histology				0.47			0.08
SCC		378/866	1.00		240/866	1.00	
Others		95/219	1.09 (0.86–1.38)		72/219	1.28 (0.97–1.68)	
Area of residence				0.06			0.50
Urban		292/707	1.00		196/707		
Rural		181/378	1.20 (0.99–1.44)		116/378	1.08 (0.86–1.37)	
Time period*				0.0001			0.06
1984–1998	No receipt	35/124	1.00		25/124	1.00	
	Receipt	298/594	1.22 (0.85–1.75)		172/594	0.90 (0.59–1.39)	
1999–2008	No receipt	51/109	1.00		37/109	1.00	
	Receipt	89/258	0.44 (0.31–0.64)		78/258	0.51 (0.34–0.78)	

*HR* hazard ratio, *SCC* squamous cell carcinoma/adenosquamous carcinoma, *No receipt* did not receive guideline treatment, *Receipt* Received guideline treatment

*p-value for interaction between time period and treatment according to the guidelines

^a^HR was adjusted for all variables shown in this table

^b^HR for patients with tumour stage IA was not considered since none died from cervical cancer in the group who were not treated according to the guidelines

**Table 6:** Effects of being treated according to the guidelines and time period on the probability of dying from all causes and cervical cancer by tumour stage (*n* = 1085)

	Any death			Death from cervical cancer		
	No. of death/Total	Adjusted HR (95 % CI)^a^	*p*-value	No. of death/Total	Adjusted HR (95 % CI)^a^	*p*-value
Stage IA^b^						
Treated according to guidelines						
No	1/54	1.00	0.08	0/54	-	
Yes	22/175	5.98 (0.80–44.83)		1/175		
Time period						
1984–1998	22/166	1.00	0.41	1/166	-	
1999–2008	1/63	0.42 (0.05–3.28)		0/63		
Stage IB-IIA						
Treated according to guidelines						
No	29/97	1.00	0.84	19/97	1.00	0.43
Yes	110/301	0.96 (0.63–1.47)		61/301	0.81 (0.48–1.38)	
Time period						
1984–1998	116/298	1.00	0.50	64/298	1.00	0.45
1999–2008	23/100	0.85 (0.53–1.37)		16/100	0.80 (0.45–1.43)	
Stage IIB-IVA						
Treated according to guidelines						
No	54/80	1.00	0.002	41/80	1.00	0.02
Yes	207/326	0.60 (0.43–0.82)		144/326	0.64 (0.44–0.93)	
Time period						
1984–1998	178/237	1.00	0.004	115/237	1.00	0.04
1999–2008	83/169	0.65 (0.48–0.87)		70/169	0.71 (0.51–0.98)	
Stage IVB						
Treated according to guidelines						
No	2/2	1.00	0.63	2/2	1.00	0.55
Yes	48/50	0.68 (0.14–3.30)		44/50	0.62 (0.13–3.02)	
Time period						
1984–1998	17/17	1.00	0.03	17/17	1.00	0.02
1999–2008	33/35	0.43 (0.20–0.93)		29/35	0.38 (0.17–0.84)	

^a^HR was adjusted for age at diagnosis, histology, area of residence, time period and treatment according to the guidelines

^b^The HR for patients diagnosed with tumours stage IA patients was not calculated because there were no deaths from cervical cancer

#### Cause-specific death

The risk of death from cervical cancer increased with tumour stage at diagnosis (*p* < 0.0001) and in women diagnosed over 65 years of age (*p* = 0.01) (Table 5). The effect of the adherence to the guidelines was found to be weakly modified by diagnosis period (*P*_*interaction*_ *= 0.06*). For those diagnosed in 1984–1998, adherence to treatment guidelines did not impact the probability of dying from cervical cancer (HR = 0.90, 95 % CI: 0.59–1.39) (Table 5). By contrast, women diagnosed in 1999–2008 and who were treated according to the guidelines had a reduced risk of dying from cervical cancer (HR = 0.51, 95 % CI: 0.34–0.78). The tumour stage stratified analysis showed independent effects of receiving treatment according to the guidelines, and time period in those with stage IIB-IVA disease (Table 6). Women with stage IIB-IVA disease who received concurrent chemo-radiotherapy, had a significantly decreased risk of death from cervical cancer (HR = 0.64, 95 % CI: 0.44–0.93, *p* = 0.02).

## Discussion

### Brief summary of the main results

A shift from radiotherapy alone to concurrent chemo-radiotherapy as the predominant treatment for IB2-IVA stage cervical cancer cases was observed since 1999 in Manitoba, Canada, which was concordant with the changes in the published treatment guidelines [4–7, 11]. The likelihood of receiving the guideline treatment, as well as the effect of guideline treatment on survival, varied by diagnosis period and tumour stage. Women diagnosed with stage IIB-IVA disease since 1999 were less likely to receive guideline treatment compared with those diagnosed earlier. This finding may to be due, in part, to older women not receiving concurrent chemo-radiotherapy because of the presence of comorbidities, poorer health or choice. The significant reduction in the risk of death from both all causes (56 %) and from cervical cancer (49 %) was observed only in those who were diagnosed since 1999 and received guideline treatment, and this appeared to be largely driven by the use of concurrent chemo-radiotherapy in stage group IIB-IVA.

### Explanation for the findings

Previous studies reported a rapid increase in the use of concurrent chemo-radiotherapy following the USA National Cancer Institute’s clinical announcement in 1999, which strongly encouraged cisplatinum-based concurrent chemo-radiotherapy for advanced stage cervical cancer [13, 14]. However, the current study observed that the change in management practice was already occurring while the related RCTs were being conducted. This implies that clinicians could have been aware of the trials from scientific meetings before the results were published in peer reviewed journals, and were ready to adopt the new evidence into the management of advanced cervical cancer. A similar phenomenon has been reported in the treatment of other cancer types, for example in the use of taxanes for primary breast cancer. The use of Paclitaxel substantially increased in the year following the presentation of study findings at the American Society of Clinical Oncology (ASCO) meeting in 1998, but the study was not published in a peer reviewed journal until five years later [20].

In agreement with previous reports, this study also found that older women were less likely to receive concurrent chemo-radiotherapy [13, 21]. Chemo-radiotherapy is associated with acute haematological, renal and gastrointestinal toxicity [22]. Therefore, patients with poor performance status and co-morbid conditions received radiotherapy alone [13]. The main reason for patients not receiving chemo-radiotherapy in Manitoba was poor renal function at diagnosis (Personal communication, Dr Robert Lotocki, CancerCare Manitoba, Canada), although detailed information was not available to adjust for treatment uptake and survival in the current analysis.

The improved survival found in this study could potentially have resulted from a range of factors, including the use of concurrent chemo-radiotherapy as well as instituting a policy of maintaining a patient’s haemoglobin to greater than 120 g/L using blood transfusion while on treatment [23]. Studies from two Canadian centres in Ontario have investigated trends in the use of concurrent chemo-radiotherapy and the resulting improved survival outcomes, although they have not included stage-specific analyses [13, 14]. An American study reported that patients who did not receive guideline treatment experienced similar survival to those who did in 1988–1994 [24]. In the current study, women who were treated according to the guidelines in 1984 and 1998 experienced the same risk of death as those who were not treated according to the guidelines. By contrast, a significant reduction in the risk of dying from all causes and from cervical cancer was observed in those who received guideline treatment from 1999 onwards. In the stratified analysis by stage, the decreased all cause and cervical cancer mortality associated with receiving recommended concurrent chemo-radiotherapy was confined to patients with stage IIB-IVA disease (40 % and 36 %, respectively), which was consistent with the relevant clinical trials and the previous studies [1–3, 21, 25–27]. The contributing effects of other treatments (such as maintenance of haemoglobin levels), which was not assessable in the current dataset, cannot be excluded.

### Strengths

This is the first population-based study of cervical cancer treatment in relation to the changes in the treatment guidelines both in Canada and internationally, which adjusted for potential confounders including tumour stage. The follow-up time in this study was longer than that previously reported in other studies with an overall median follow up time of 6.4 years [25].

### Limitations

Similar to other population-based studies using administrative data [14, 25], this study has some limitations related to data availability. Therefore, we could only assess broad concordance with guidelines and could not take into account individual patient factors, specifically the effects of co-morbidities, poor performance status and patients’ treatment preferences. Similarly, we could not assess other underlying factors related to practice that deviated from the guidelines, such as clinician referral practice, limitations in health care access and patient compliance. Accordingly, we are unsure to what extent each of these non-assessable factors contributed to the suboptimal treatment patterns observed. This is an area for future research if information on patients’ co-morbidity and performance status, such as the Eastern Cooperative Oncology Group (ECOG) score, is routinely recorded in the clinical charts. About 20 % of cervical cancer patients diagnosed during the study period in Manitoba did not have their tumour stage recorded, and therefore were not included in the analysis. Missing stage information for these patients may have had an effect on the overall treatment patterns in accordance with the guidelines. Nevertheless, the extensive record linkage system in Manitoba allowed us to obtain demographic information for all patients as well as detailed information on treatment and stage for the majority (77 %) of cervical cancer cases over a long period of time, and we were thereby able to examine the effect of guideline-recommended treatment on survival after adjusting for confounding effects due to age and stage of disease.

## Conclusions

Consistent evidence from clinical trials investigating the survival benefit of concurrent chemo-radiotherapy on cervical cancer led to substantial revision of treatment guidelines. We found this resulted in a rapid increase in the use of concurrent chemo-radiotherapy and an associated significantly increased survival in women diagnosed with invasive stage IIB-IVA cervical cancer, although the effect of other changes in clinical practice on increased survival cannot be ruled out.

## Abbreviations

FIGO: International Federation of Gynecology and Obstetrics
MCR: Manitoba Cancer Registry
HR: Hazard ratio
CI: Confidence interval
AJCC: American Joint Committee on Cancer
